# Impaired consciousness is linked to changes in effective connectivity of the posterior cingulate cortex within the default mode network

**DOI:** 10.1016/j.neuroimage.2015.01.037

**Published:** 2015-04-15

**Authors:** Julia Sophia Crone, Matthias Schurz, Yvonne Höller, Jürgen Bergmann, Martin Monti, Elisabeth Schmid, Eugen Trinka, Martin Kronbichler

**Affiliations:** a Neuroscience Institute & Centre for Cognitive Neuroscience, Christian Doppler Klinik, Paracelsus Medical University, Salzburg, Austria; b Centre for Cognitive Neuroscience & Department of Psychology, University of Salzburg, Salzburg, Austria; c Department of Neurology, Christian Doppler Klinik, Paracelsus Medical University, Salzburg, Austria; d Department of Psychology, University of CA Los Angeles, USA

**Keywords:** Disorders of consciousness, Effective connectivity, Vegetative state, Dynamic causal modeling, Default mode network, Posterior cingulate cortex

## Abstract

The intrinsic connectivity of the default mode network has been associated with the level of consciousness in patients with severe brain injury. Especially medial parietal regions are considered to be highly involved in impaired consciousness. To better understand what aspect of this intrinsic architecture is linked to consciousness, we applied spectral dynamic causal modeling to assess effective connectivity within the default mode network in patients with disorders of consciousness.

We included 12 controls, 12 patients in minimally conscious state and 13 in vegetative state in this study. For each subject, we first defined the four key regions of the default mode network employing a subject-specific independent component analysis approach. The resulting regions were then included as nodes in a spectral dynamic causal modeling analysis in order to assess how the causal interactions across these regions as well as the characteristics of neuronal fluctuations change with the level of consciousness.

The resulting pattern of interaction in controls identified the posterior cingulate cortex as the main driven hub with positive afferent but negative efferent connections. In patients, this pattern appears to be disrupted. Moreover, the vegetative state patients exhibit significantly reduced self-inhibition and increased oscillations in the posterior cingulate cortex compared to minimally conscious state and controls. Finally, the degree of self-inhibition and strength of oscillation in this region is correlated with the level of consciousness.

These findings indicate that the equilibrium between excitatory connectivity towards posterior cingulate cortex and its feedback projections is a key aspect of the relationship between alterations in consciousness after severe brain injury and the intrinsic functional architecture of the default mode network. This impairment might be principally due to the disruption of the mechanisms underlying self-inhibition and neuronal oscillations in the posterior cingulate cortex.

## Highlights

• In controls, the posterior cingulate cortex is the main driven hub within the default mode network.
• In impaired consciousness, this pattern of interaction is disrupted.
• The posterior cingulate cortex has lost its inhibitory regulation mechanisms.
• Impaired consciousness may be due to disruption in these regulation mechanisms.
• Findings emphasize the critical role of this posterior region in consciousness.

## 1 Introduction

Understanding alterations in intrinsic connectivity networks of severe brain injury is essential for clinical purposes (Sharp et al., 2014). Especially for the challenging assessment in disorders of consciousness (Schnakers et al., 2009), that is, patients in vegetative state/unresponsive wakefulness syndrome (Laureys et al., 2010) (VS/UWS) and minimally conscious state (MCS), resting-state fMRI is a powerful tool. Patients with disorders of consciousness are awake but not or only minimal aware of their environment, therefore, showing a dissociation between awareness and arousal. For this reason, they provide the unique opportunity to investigate alterations in brain processing directly related to impaired consciousness.

Previous studies revealed dysfunctional connectivity of the default mode network (DMN) in disorders of consciousness (Boly et al., 2009), and MCS patients displaying a more preserved pattern of network connectivity as compared to VS/UWS patients (Crone et al., 2013; Fernandez-Espejo et al., 2010; Kotchoubey et al., 2012; Vanhaudenhuyse et al., 2010). Moreover, deactivation of the DMN is associated with the level of consciousness in patients (Crone et al., 2011) and cognitive performance in healthy volunteers (Bonnelle et al., 2012). Additionally, DMN connectivity has also been shown to have prognostic value for comatose patients (Norton et al., 2012). Within this network, medial parietal regions and their connectivity with medial frontal regions have been shown to be critically involved in alterations of consciousness after severe brain injury (Crone et al., 2013; Fernandez-Espejo et al., 2011; Laureys et al., 1999; Vanhaudenhuyse et al., 2010), during sleep (Horovitz et al., 2009; Horovitz et al., 2008; Samann et al., 2011) and during propofol-induced sedation (Boly et al., 2012; Fiset et al., 1999; Monti et al., 2013).

However, most studies addressing the intrinsic function of the DMN in altered states of consciousness have relied upon a functional connectivity approach. While productive, the simple observation of patterns of correlations between distant regions over time does not provide any insight into the causal organization underlying the observed correlations (Friston, 1994). In the present research, we therefore adopt an effective connectivity approach to identify the causal interactions between regions within the DMN, thereby allowing a much deeper understanding of the alterations in the functional brain architecture underlying disorders of consciousness. Effective connectivity within the DMN has been investigated in healthy volunteers employing DCM for resting-state fMRI (Di and Biswal, 2013; Li et al., 2012). The findings highlight the role of the posterior cingulate cortex (PCC) as a central hub within the DMN confirming findings in previous studies proposing the PCC as a main connector hub between distinct networks (Hagmann et al., 2008; van den Heuvel and Sporns, 2011).

Recently, a new DCM method for resting-state fMRI has been introduced especially suited for group comparisons. Spectral DCM is based upon a deterministic model that generates predicted crossed spectra which allows to asses effective connectivity engendered by the underlying functional connectivity (Friston et al., 2014). The advantage of this approach lies in its computational efficiency and, more importantly, it also provides the opportunity to compare , in addition to effective connectivity, the characteristics of, the neuronal fluctuations across groups.

In the present study, we investigated the direction of coupling strength and specific properties of neuronal fluctuations within the DMN in patients with disorders of consciousness using spectral DCM. We hypothesize that the effective connectivity of the PCC and its role as a driven hub is altered in patients and that this alteration is related to the level of consciousness.

## 2 Materials and methods

The study was approved by Ethics Commission Salzburg (Ethikkommission Land Salzburg; number 415-E/952).

### 2.1 Participants

In this study, 15 patients in MCS, 17 patients in VS/UWS, and 13 age-matched healthy controls with no history of neurological or psychiatric disease were investigated. This small sample of 32 patients was selected based on the criteria of repeatedly examined and unambiguously diagnosed cases. Participants were scanned at the Neuroscience Institute, Christian-Doppler-Klinik, Paracelsus Medical University, Salzburg. From this sample, we only included subjects in further analyses with movement parameters smaller than 3 mm translation and 3° rotation. Moreover, we carefully controlled image realignment and segmentation by visual inspection and only included those patients for which realignment and segmentation has been successful. In consequence, three patients in MCS, four patients in VS/UWS, and one control subject were excluded resulting in a sample of 12 healthy controls (mean age = 55; age range = 44–70; 8 female), 12 patients in MCS (mean age = 51; age range = 28–71; 6 female) and 13 patients in VS/UWS (mean age = 54; age range = 32–73; 3 female). All patients participating in this study were examined with the Coma Recovery Scale — Revised (CRS-R) (Giacino et al., 2004) in a weekly interval during in-patient stay. Classification of patients based on the diagnosis obtained with the CRS-R at time of scanning. All patients showed preserved auditory functioning, largely preserved brainstem reflexes, and a fairly preserved sleep–wake-cycle as assessed by neurological examination. None of the patients were artificially ventilated or sedated at time of scanning. Additional information of the individual patients is listed in the Inline Supplementary Table S1. Written informed consent was obtained from all healthy subjects and from the guardianship of all patients according to the Declaration of Helsinki.

In this study, 15 patients in MCS, 17 patients in VS/UWS, and 13 age-matched healthy controls with no history of neurological or psychiatric disease were investigated. This small sample of 32 patients was selected based on the criteria of repeatedly examined and unambiguously diagnosed cases. Participants were scanned at the Neuroscience Institute, Christian-Doppler-Klinik, Paracelsus Medical University, Salzburg. From this sample, we only included subjects in further analyses with movement parameters smaller than 3 mm translation and 3° rotation. Moreover, we carefully controlled image realignment and segmentation by visual inspection and only included those patients for which realignment and segmentation has been successful. In consequence, three patients in MCS, four patients in VS/UWS, and one control subject were excluded resulting in a sample of 12 healthy controls (mean age = 55; age range = 44–70; 8 female), 12 patients in MCS (mean age = 51; age range = 28–71; 6 female) and 13 patients in VS/UWS (mean age = 54; age range = 32–73; 3 female). All patients participating in this study were examined with the Coma Recovery Scale — Revised (CRS-R) (Giacino et al., 2004) in a weekly interval during in-patient stay. Classification of patients based on the diagnosis obtained with the CRS-R at time of scanning. All patients showed preserved auditory functioning, largely preserved brainstem reflexes, and a fairly preserved sleep–wake-cycle as assessed by neurological examination. None of the patients were artificially ventilated or sedated at time of scanning. Additional information of the individual patients is listed in the Inline Supplementary Table S1. Written informed consent was obtained from all healthy subjects and from the guardianship of all patients according to the Declaration of Helsinki.

Table S1. Patients’ information

**Table S1:** Patients’ information.

Subjects	Age	Etiology	Time since onset (in days)	Auditory function	Visual function	Motor function	Verbal/Oromotor function	Communication	Arousal
*MCS*									
MCS01	28	Limbic encephalopathy	148	None	Visual pursuit	Flexion withdrawal	Oral reflexive movement	None	Without stimulation
MCS02	63	Subarachnoidal hemorrhage	62	Auditory startle	Object localization: Reaching	Flexion withdrawal	None	None	Without stimulation
MCS03	62	Subarachnoidal hemorrhage	74	Localization to sound	Visual pursuit	Flexion withdrawal	Oral reflexive movement	None	Attention
MCS04	46	Multiple cerebral infarct	41	Auditory startle	Visual startle	Automatic motor response	Oral reflexive movement	None	With stimulation
MCS05	65	Cardiopulmonary resuscitation	85	Localization to sound	Fixation	Object manipulation	Oral reflexive movement	None	Attention
MCS06	31	Traumatic brain injury	66	None	None	Localization to noxious stimuli	Oral reflexive movement	None	With stimulation
MCS07	52	Subarachnoidal hemorrhage	146	Localization to sound	Visual startle	Localization to noxious stimuli	Oral reflexive movement	None	Without stimulation
MCS08	61	Intracerebral hemorrhage	75	None	Visual pursuit	Localization to noxious stimuli	Oral reflexive movement	None	With stimulation
MCS09	53	Status epilepticus	49	None	None	Flexion withdrawal	Oral reflexive movement	None	Without stimulation
MCS10	51	Status epilepticus	135	None	Visual pursuit	Flexion withdrawal	Oral reflexive movement	None	Without stimulation
MCS11	71	Subarachnoidal hemorrhage	70	None	Visual pursuit	Flexion withdrawal	Oral reflexive movement	None	With stimulation
MCS12	32	Multiple cerebral infarct	37	None	Visual pursuit	Abnormal posturing	Vocalization/Oral movement	None	With stimulation

*VS*									
VS01	32	Basilaris thrombosis	922	Auditory startle	None	Flexion withdrawal	Oral reflexive movement	None	With stimulation
VS02	66	Traumatic brain injury & subarachnoidal hemorrhage	82	None	Visual startle	Flexion withdrawal	None	None	Without stimulation
VS03	36	Cardiopulmonary resuscitation	66	Auditory startle	None	Abnormal posturing	Oral reflexive movement	None	Without stimulation
VS04	55	Cardiopulmonary resuscitation	82	None	Visual startle	None	Oral reflexive movement	None	With stimulation
VS05	54	Cardiopulmonary resuscitation	70	Auditory startle	Visual startle	None	None	None	With stimulation
VS06	35	Status epilepticus	49	None	None	Flexion withdrawal	Oral reflexive movement	None	Without stimulation
VS07	59	Subarachnoidal hemorrhage	20	Auditory startle	None	Flexion withdrawal	Oral reflexive movement	None	With stimulation
VS08	55	Cardiopulmonary resuscitation	186	Auditory startle	Visual startle	Abnormal posturing	Oral reflexive movement	None	With stimulation
VS09	73	Traumatic brain injury	59	None	None	Abnormal posturing	Oral reflexive movement	None	With stimulation
VS10	60	Traumatic brain injury	60	None	None	Flexion withdrawal	Oral reflexive movement	None	With stimulation
VS11	43	Cardiopulmonary resuscitation	68	Auditory startle	None	Abnormal posturing	Oral reflexive movement	None	Without stimulation
VS12	52	Traumatic brain injury & status epilepticus	64	Auditory startle	Visual startle	Abnormal posturing	Vocalization/Oral movement	None	With stimulation
VS13	82	Cardiopulmonary resuscitation	27	None	None	Abnormal posturing	Vocalization/Oral movement	None	Without stimulation

MCS = patients in minimally conscious state; VS = patients in vegetative state (unresponsive wakefulness syndrome).

Inline Supplementary Table S1

Inline Supplementary Table S1 can be found online at http://dx.doi.org/10.1016/j.neuroimage.2015.01.037.

### 2.2 Data acquisition

Resting-state fMRI data were obtained using a three Tesla Siemens TIM TRIO (Siemens AG, Munich, Germany; 250 T2*-weighted images were obtained in descending order; 36 slices with 3 mm thickness; FoV = 192 mm^2^; TR = 2250 ms; TE = 30 ms; flip angle = 70°; matrix size = 64 × 64). Subjects were instructed to let their thoughts run free and not to think about anything special. In addition, high-resolution, T1-weighted MP-RAGE sequences (160 slices; slice thickness = 1.2 mm; TR = 2300 ms; TE = 2.91 ms; voxel size = 1 × 1 × 1.2 mm; FoV = 256 mm^2^; flip angle = 9°) for anatomic information were acquired for each participant.

### 2.3 Preprocessing

Functional data were preprocessed using Statistical Parametric Mapping (version SPM8; http://www.fil.ion.ucl.ac.uk/spm/). The first six functional scans were considered as dummy scans and were discarded. Preprocessing steps included the following procedures: segmentation of the T1-weighted image to compute the gray matter images; realignment to compensate for motion; unwarping; slice timing correction; coregistration of the mean echo planar imaging (EPI) to the participant's own anatomical scan; affine-only normalization to standard stereotaxic anatomical MNI space; data were spatially smoothed using a Gaussian Kernel of 8 mm FWHM. Voxel size was resampled to 3 × 3 × 3 mm. Note that affine-only normalization (i.e., no nonlinear functions) was performed because of the partially severe and wide-spread lesions in the patients' brains.

We additionally assessed the framewise displacement calculated from derivatives of the six rigid body realignment parameters and the root mean squared change in BOLD signal from volume to volume (DVARS) (Power et al., 2012) using FSL (Jenkinson et al., 2002). Framewise displacement and DVARS values were compared between the three groups using One-way ANOVA with group as a factor. There were no significant differences between groups (FD: *F* = 1.36, *p* = 0.272; DVARS: *F* = 2.18, *p* = 0.129).

### 2.4 Selection of regions of interest

The same four regions of the DMN (medial frontal cortex (MFC); PCC; lateral inferior parietal lobules (IPL)) as in previous analyses using DCM (Bastos-Leite et al., 2014; Di and Biswal, 2013; Li et al., 2012) were selected as regions of interest (ROIs). To identify the coordinates, independent component analysis (ICA) was performed for each of the three groups using the Group ICA of fMRI Toolbox (GIFT) (http://icatb.sourceforge.net/). GICA3 was used for back-reconstruction type and 20 components were extracted. The resulting components were spatial correlated with a template image of a meta-analysis of DMN functional heterogeneity (Laird et al., 2009) and verified by visual inspection. The coordinates were extracted from each individual independent component for each of the four regions using Talairach Daemon software and icbm2tal transform as implemented in GIFT and then transformed into MNI space using GingerALE software Version 2.3.2. See Table e-2 for coordinates of each region in each participant. This procedure ensures that DCM analysis is performed on those regions identified as functionally connected within every individual DMN. This is particularly important because in patients with severe brain injury the anatomical and functional organization of brain regions may be altered which may lead to a mis-specification of regional activity when extracting data from coordinates based on normalized brain atlases.

### 2.5 General linear model

The general linear model (GLM) included the six rigid body realignment parameters to account for head motion. The white matter and cerebrospinal fluid mean signals were also included into the model as regressors. In addition, the GLM contained an implicit high-pass filter of 1/100 Hz to remove possible ultraslow fluctuations due to hardware related drift.

### 2.6 Dynamic causal modeling

**Fig. 1:**
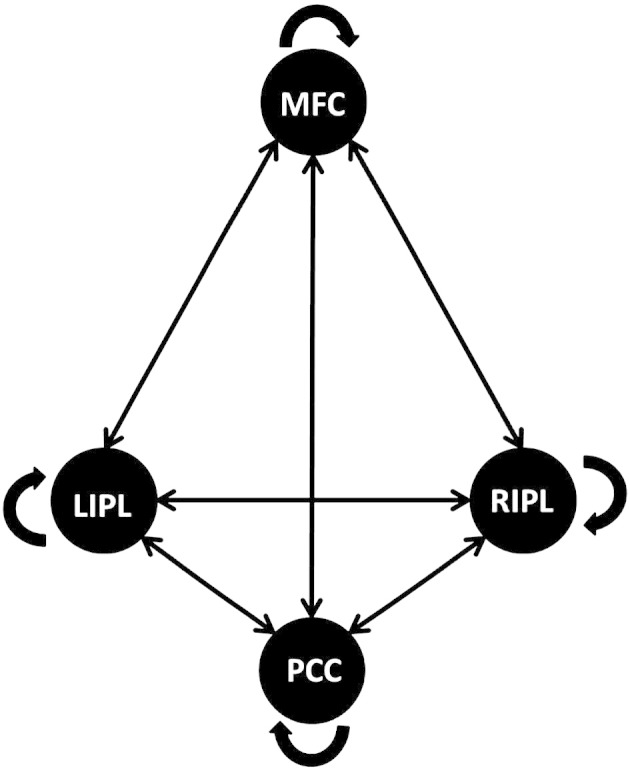
Schematic representation of the fully and reciprocally connected model. All possible connections are displayed between the medial frontal cortex (MFC), the posterior cingulate cortex (PCC), the left inferior parietal lobule (lIPL), and the right inferior parietal lobule (rIPL).

DCM was performed by following the steps detailed below: (1) extraction of BOLD fMRI time series from each subject using the individual coordinates; (2) specification of the model space based on a fully and reciprocally connected model (see Fig. 1); (3) estimation of the specified model; (4) implementation of a post-hoc model selection routine.

DCM analyses were performed with the DCM12 routine implemented in SPM12 (Wellcome Department of Cognitive Neurology, London, UK; http://www.fil.ion.ucl.ac.uk/spm/). To extract the BOLD fMRI time series, volumes of interest were defined as spheres with a radius of 8 mm centered at the individual coordinates of each subject. Note that the first eigenvectors were extracted after modeling the GLM removing effects of head motion and low-frequency drift.

The specified model was the full and reciprocal connected model as shown in Fig. 1. The connections between the four regions were specified as fixed connections including the recurrent connection of each node (matrix A). This model was fitted with an estimation procedure using second-order statistics characterizing spectral densities over frequencies, that is, complex cross spectra. Instead of predicting the times-series itself, spectral DCM uses the Fourier transform of the cross correlation of the time series as a data feature for prediction. For details see Friston et al. (2014). We used this new deterministic approach in effective connectivity for investigating differences between groups because it is not restricted to the comparison of connection strength, but also provides the opportunity to look at differences in amplitude and exponent of the neuronal fluctuations (that is, the strength and frequencies of oscillations).

Inferences about directed connectivity can proceed at two levels; either at the level of the model or at the level of connection strengths (parameters) under a given model. In what follows, we will pursue analyses at both the level of models and parameters. Put simply, a difference in models between groups implies that one or more connections are absent in a quantitative sense; whereas a difference in the parameters (effective connectivity) suggest that the random effects of subjects are expressed qualitatively in terms of the connection strengths, under the assumption that the connection exists.

To explore all possible dynamic causal models, a post-hoc model selection routine (Friston and Penny, 2011) was applied to determine the best fitting model for each group. This approach fits the full model with all its free parameters to the given data. In a next step, the evidence for all reduced models, that is, all possible models nested in the full model, is approximated by effectively removing the parameters. It should be noted that with more than 8 parameters, the post-hoc model selection routine implements a “greedy search” over all models formed by removing all permutations of the 8 parameters whose individual removal produced the smallest reduction in model evidence (as computed using the Savage–Dickey or post-hoc approximation) resulting in 2^8^ = 256 reduced models. This post-hoc procedure achieves very similar results as the conventional Bayesian model selection but is much more efficient when comparing a large model space (Friston and Penny, 2011; Rosa et al., 2012). Results of the model selection procedure are shown as a model posterior; the probability of a particular model being the best compared to any other model given the group data. For inference at the parameter space level, it is necessary that parameters are compared within the same winning model (Seghier et al., 2010) to exclude the possibility that between-group differences in estimated parameters may be due to differences in model fit.

### 2.7 Parameter estimations

In a next step, we investigated the effective connectivity at the individual parameter level to identify differences between various groups. All parameters of intrinsic connectivity (DCM.Ep.A) as well as the values of the amplitude and exponent of neuronal fluctuations (DCM.Ep.a) were compared between groups using ANOVA permutation tests implemented in the lmPerm library in R (www.R-project.org) with group as a factor. Post-hoc tests (additionally corrected for false-discovery rate) were applied to all significant results. We also performed a Spearman correlation analysis for the patients' data between the CRS-R scores and those surviving parameters of effective connectivity and neural activity, showing significant differences between groups after correction for multiple comparisons to explore whether the responsiveness of a patient is associated with the connectivity strength or the form and amplitude of neuronal fluctuations within the DMN. All *p*-values were corrected for false-discovery rate. Moreover, since permutation testing is a nonparametric test, in which the false positive rate is exactly equal to the specified α level, we expect less than 1 false positive at *p* < 0.042 uncorrected, given the 24 ANOVAs performed (that is, 12 comparisons with the intrinsic connectivity between the 4 nodes, 4 comparisons with the recurrent connectivity within the 4 nodes and 8 comparisons with the form and amplitude of neuronal fluctuations in each node).

## 3 Results

### 3.1 Post-hoc model selection

**Fig. 2:**
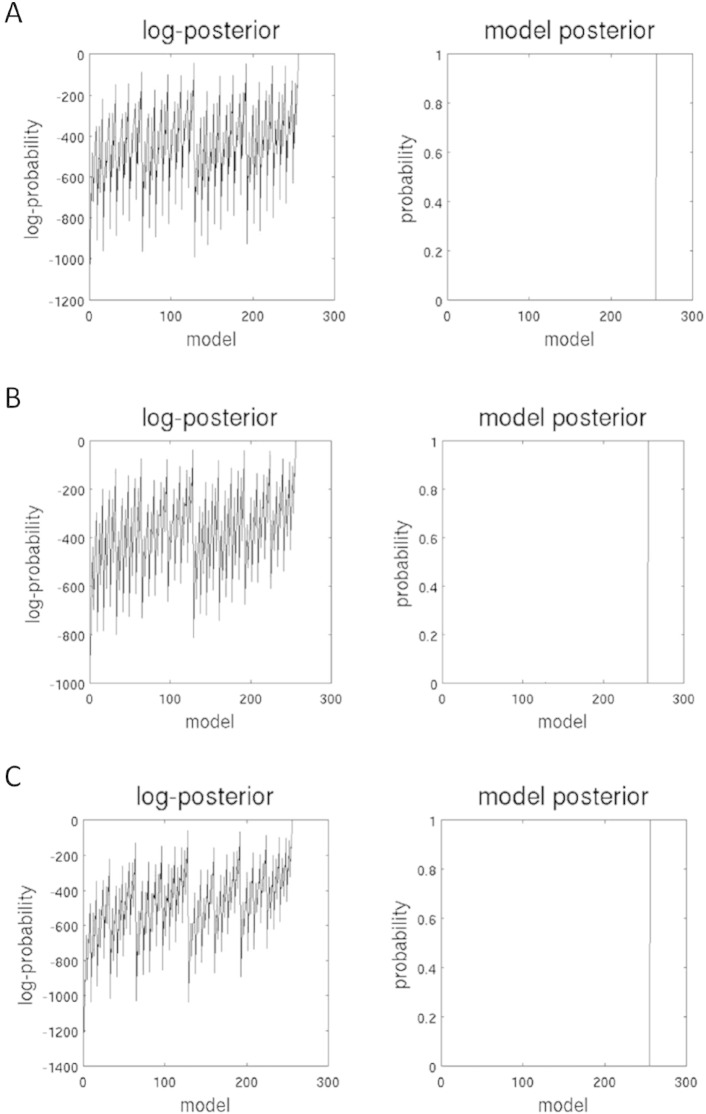
Results of the post-hoc model selection procedure. The two columns represent the log-posterior and model posterior probabilities of all evaluated models examined for healthy controls (A), patients in minimally conscious state (B), and patients in vegetative state (C). The full model is the winning model in each group with a posterior probability of almost 1.

Post-hoc model selection compared the evidence of all investigated models for each group. In all three groups, the procedure revealed the fully connected model as the ‘winning’ model with a probability of almost 1 (see Fig. 2). The fully connected model has 16 free parameters describing the intrinsic connections between nodes and the recurrent intrinsic connections within nodes, respectively. In Fig. 2, the profile of model evidences are shown with the posterior probability for each model. In all three groups, the full model (model nr. 256) has a probability of almost 1 and a log-probability of almost 0. The next best model (model nr. 128) has a very low probability with almost 0 for all three groups and a log-probability of − 43.6 for the control group, − 36.2 for the MCS group, and − 57.4 for the VS/UWS group. To estimate the significance of this result, the Bayes factor was calculated by dividing the probability of the ‘winning’ model (almost 1) by the probability of the second most probable model (almost 0) suggesting very strong evidence for the winning model for each group since a Bayes factor of 3:1 is still considered as positive evidence (Kass and Raftery, 1995).

### 3.2 Parameter estimates

After identifying the most probable model, the specific properties of effective connectivity within the DMN were explored at the parameter level, that is, connectivity strength of the intrinsic connections within and between nodes, as well as characteristics of oscillations.

**Fig. 3:**
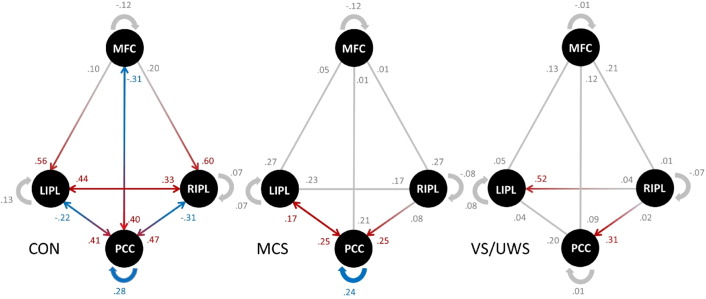
Significant connections within the full model in each group. Strength and direction of connectivity between the medial frontal cortex (MFC), the posterior cingulate cortex (PCC), the left inferior parietal lobule (lIPL), and the right inferior parietal lobule (rIPL) are displayed for healthy controls (CON), patients in minimally conscious state (MCS) and patients in vegetative state (VS/UWS). Red arrows and values indicate positive coupling, blue arrows and values indicate negative coupling. Gray values represent connections which did not differ significantly from zero in each group. In healthy volunteers, the PCC appears to be the main driven hub receiving positive input and giving negative feedback. In contrast, this pattern of interaction seems to be disrupted in patients.

Fig. 3 displays the connectivity strength and direction of each connection between and within the four nodes for all three groups. Red and blue arrows (and values) represent those connections which are significantly different from zero in each group, while red arrows represent a positive connection strength and blue arrows a negative connection strength. Please note that the recurrent connectivity strength within each node, indicated with blue arrows, can have negative *and* positive values even though the connections strength is negative. This is because recurrent connections are assumed to always be inhibitory on biological constraints. In SPM12, the parameterization of the self-connections are log-scaled and the prior expectation is fixed at − 0.5 (with − 0.5 ∗ exp(A); where A has a prior mean of 0 and − 0.5 ∗ exp(0) = − 0.5). Consequently, a positive value means that the self-connection is more inhibitory and a negative value means that it is less inhibitory compared to the prior. In healthy controls, almost all reciprocal connections (except from the lateral IPL to the MFC) are significant. Interestingly, all driving influence from the PCC is negative. In patients, only a few connections within the full model are significant and none of the connections between nodes are negative.

**Fig. 4:**
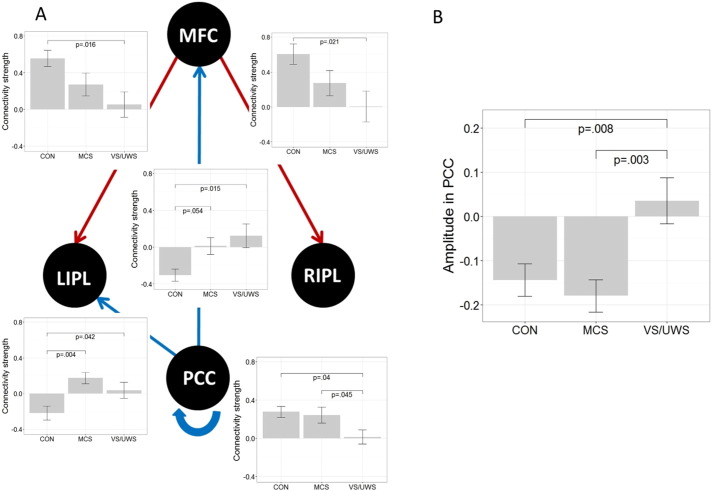
Significant differences in parameter estimates between groups. (A) Significant differences between healthy controls (CON), patients in minimally conscious state (MCS) and patients in vegetative state (VS/UWS) in the strength of effective connectivity between the medial frontal cortex (MFC), the posterior cingulate cortex (PCC), the left inferior parietal lobule (lIPL), and the right inferior parietal lobule (rIPL). Red arrows indicate positive values, blue arrows indicate negative values for controls. Note that means for the recurrent connectivity strength are inhibitory with positive values indicating a stronger effect relative to prior. (B) Significant differences between groups in the amplitude of neuronal fluctuations of the posterior cingulate cortex. Bar-plots represent means and standard errors; all *p*-values are corrected for multiple comparisons.

Connectivity strength (DCM.Ep.A) as well as characteristics in the oscillation of neuronal fluctuations (DCM.Ep.a) were compared between groups using ANOVA permutation testing with additional post-hoc tests. Significant differences between groups in the connectivity strength are displayed in Fig. 4A. All reported *p*-values are corrected for false-discovery rate. In addition, please note that all significant results from the 24 ANOVAs performed with permutation testing were significant at *p* < 0.04151, thus, we expect less than 1 false positive (see Materials and methods section).

One of the most important results is that the strength of the self-connection within the PCC differs between patients; it is lower in VS/UWS patients compared to MCS patients (see Fig. 4A). The VS/UWS patients also exhibit lower strength of inhibitory self-connectivity in the PCC than healthy controls, while MCS patients and healthy controls do not differ. Negative connectivity strength from the PCC to the MFC was stronger in the control group compared to MCS and VS/UWS (while only the difference between controls and VS/UWS is significant at *p* < 0.05). In addition, negative connectivity strength from the PCC to the left IPL was significantly stronger in the control group compared to MCS and VS/UWS. Positive connectivity strength from the MFC to both lateral IPL was significantly stronger in the control group compared to VS/UWS. We also observed the trend that the connectivity strength of the efferent connections from the MFC are lower in MCS compared to controls and going towards zero in VS/UWS. However, this trend (for the connection with the PCC in particular) is not significant at a corrected level.

Our second important finding is a significant difference between patients in the amplitude of neuronal fluctuations in the PCC (Fig. 4B) showing that MCS as well as healthy controls exhibit less stronger oscillations to drive the neuronal response compared to the VS/UWS.

**Fig. 5:**
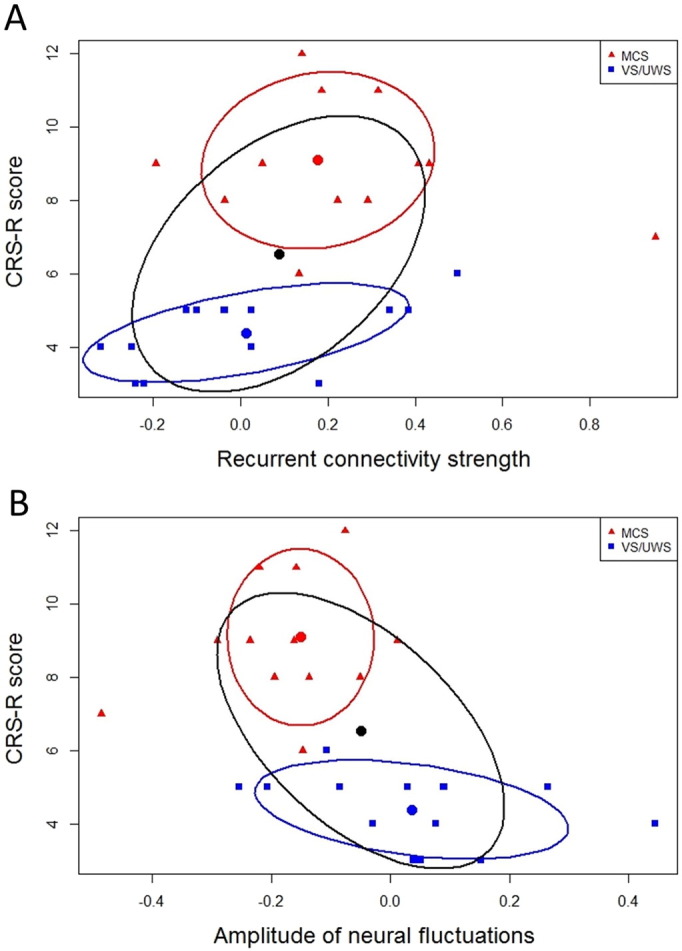
Significant correlation between behavioral responsiveness of patients and parameter estimates of the posterior cingulate cortex. (A) Significant correlation between behavioral responsiveness (CRS-R scores) and recurrent connectivity strength of the posterior cingulate cortex for patients in minimally conscious state (MCS) and patients in vegetative state (VS/UWS). (B) Significant correlation between CRS-R scores and amplitude of neuronal fluctuations of the posterior cingulate cortex. Correlations were performed using Spearman's Rho. The black confidence ellipse indicates correlation strength for both groups together. The red and blue confidence ellipses indicate correlation strength for each patient group, respectively.

To further explore the relation of behavioral responsiveness and properties of effective connectivity in patients, a Spearman correlation (two-tailed) was calculated between the CRS-R scores and those values showing significant results in the ANOVA. All *p*-values were corrected for false-discovery rate. Recurrent connectivity in the PCC correlates positively with behavioral performance in patients (*r* = .48, *p* = 0.044). Moreover, the amplitude of neuronal fluctuations in the PCC correlates negatively with the CRS-R scores of each patient (*r* = − .58, *p* = 0.015). Fig. 5 displays the correlation over both groups and for each group separately. The overall significant correlation is reflected in the VS/UWS but not in the MCS group.

## 4 Discussion

Identifying specific alterations in network interaction after severe brain injury and relating them to specific impairments such as disorders of consciousness has important implications for the clinical setting but also for brain research in general (Sharp et al., 2014). Decreased functional (Boly et al., 2009; Crone et al., 2013; Vanhaudenhuyse et al., 2010) as well as structural (Fernandez-Espejo et al., 2012) connectivity of medial parietal regions in relation to the degree of impairment have been already demonstrated by previous studies of spontaneous brain function in disorders of consciousness. The present work is the first to explore the relationship between causal interactions of medial parietal regions within the DMN and disorders of consciousness by employing DCM for resting-state fMRI.

Overall, we have reported two main findings. First, healthy volunteers demonstrate a DCM architecture in which the PCC is the target of strong positive driving input from the anterior and lateral nodes (MFC and IPL, respectively), and the source of negative driving output towards the anterior and lateral nodes. In patients, however, the overall strength of directed connectivity among regions in the DMN is relatively low, and the PCC no longer appears to serve as the main driven hub. On the one hand, all efferent connections from anterior to parietal regions clearly show a trend towards lower influence in minimally responsive patients and almost zero influence in unresponsive patients compared to healthy volunteers. On the other hand, the efferent connections of the PCC change their type of influence from positive to negative across groups (see Figs. 3 and 4). Negative values of connectivity strength in DCM for resting-state fMRI, that is, the rate constants of neuronal responses measured in Hz, have been typically interpreted as inhibitory (Friston et al., 2014; Li et al., 2012). Provided that all true influences on the specific regions have been captured by the specified DCM model, the PCC in healthy volunteers receives excitatory input from all other nodes while its feedback is inhibitory. In unconscious and minimal conscious patients, however, this inhibitory feedback is nonexistent. We note that our usage of the term ‘feedback’ differs from the meaning typically employed to describe sensory processing. In our context, this term does not imply a hierarchical structure but rather stresses the presence of reciprocal connectivity between DMN nodes.

The critical role of the PCC in disorders of consciousness becomes even more evident when considering the second finding which shows differences between patient groups. VS/UWS patients exhibit reduced strength of recurrent (inhibitory) connectivity and stronger oscillations within the PCC as compared to the MCS patients and the control group. These two characteristics of effective connectivity (i.e., strength of recurrent connectivity and oscillations) in the PCC are also significantly associated with clinical (i.e., behavioral) measures of consciousness after severe brain injury in both patient groups. As conscious behavior increases, down-regulation of the PCC itself gets more effective, while (perhaps as a consequence) the amplitude of neuronal fluctuations decreases. To avoid a circularity effect driven by the ANOVA group difference, it is important to also look at this relationship for each group separately. As can be seen in Fig. 5, the overall association between behavioral measures of consciousness and the two characteristics of effective connectivity is reflected in the VS/UWS group. In the MCS group, however, it can barely be described as a trend possibly due to the high variability in this group. The lack of inhibitory (self-) regulation of the PCC may be a reason for the increased oscillations observed in the PCC in unresponsive patients. However, since this connectivity analysis is based on the BOLD response, we are not able to disentangle the relationship between oscillation strength driving neuronal responses and the inhibitory or excitatory influence of this region. All we may conclude is that the BOLD response of one specific region is influencing the BOLD response of another region in a positive or negative manner, under the assumption, and this is important, that the specified model is a closed system in the sense that all influences are actually modeled.

It should be noted that, to date, differences in functional connectivity between MCS and VS/UWS, that is, between minimal consciousness and unconsciousness, have been pinpointed reliably and exclusively to medial parietal regions such as the PCC. Medial parietal regions belong to the so-called ‘rich club’ representing regions which are more inter-connected throughout the brain than other nodes. They are suggested to play a critical role in overall brain communication by enabling highly efficient information integration (van den Heuvel and Sporns, 2011). The PCC is a major transit hub for exchange of information throughout the whole brain (Deshpande et al., 2011; Hagmann et al., 2008; Honey et al., 2009; Yan and He, 2011). Recent studies reveal a complex pattern of interaction with different functional connectivity networks emphasizing a multifaceted role of the PCC in global brain communication (Leech et al., 2012; Leech et al., 2011). One main theory proposes that the PCC plays a critical role in conscious internally-directed thought (Binder et al., 1999; Mason et al., 2007; Raichle and Snyder, 2007). A more complex role of the PCC in cognition has been recently postulated by Leech and Sharp (2014). The authors propose an integrated Arousal-Balance-and-Breadth-of-Attention model which assumes that the PCC is sensitive to the state of arousal but serves also as a complex control mechanism of attention.

The differences between patient groups regarding the functionality of the PCC cannot be simply due to differences in arousal since patients with disorders of consciousness show a dissociation of arousal and awareness (Laureys et al., 2004). Indeed, while they both have recovered from coma to a similar degree of wakefulness, they do differ in the level of conscious awareness. With this in mind, it is reasonable to conclude that the observed alterations of the PCC are directly involved in the level of impaired consciousness.

We also observed (non-significant) differences in the inter-hemispheric connectivity between groups, as can be seen in Fig. 3. While in healthy controls the inter-hemispheric connectivity is reciprocal, which replicates findings of previous studies using DCM for resting-state fMRI (Bastos-Leite et al., 2014; Li et al., 2012; Razi et al., 2014), there seems to be an imbalance of inter-hemispheric connectivity in VS/UWS. However, this finding should be interpreted with caution since the differences are not significant and interpretation of inter-hemispheric asymmetry is confounded by unilateral lesions.

There are some inconsistencies with previous studies investigating effective connectivity of the DMN regarding findings in healthy volunteers. A majority of these studies identified influences from the MFC to the PCC and not vice versa. However, this may be due to differences in the methodological approaches such as Granger causality (Jiao et al., 2011; Uddin et al., 2009; Zhou et al., 2011) and deterministic DCM (Di and Biswal, 2013) which are perhaps less appropriate for resting-state fMRI (see discussion below). Moreover, two of the three studies employing DCM for resting-state fMRI (stochastic and spectral DCM) did not use subject-specific coordinates to specify the DMN nodes (Bastos-Leite et al., 2014; Razi et al., 2014). However, in the face of structural and functional variability across subjects, it is very important to identify nodes at an individual level to ensure appropriate selection of coordinates. Bastos-Leite et al. (2014), for example, performed Group ICA on data of an independent control group to obtain the coordinates of the four DMN nodes. This may also be an explanation for the very low connectivity strength between regions reported in their work. Likewise, Razi et al. (2014) specified the DMN nodes using a standard seed-based functional connectivity analysis at group level. The authors themselves acknowledge that interpretation of these empirical results is therefore limited. Remarkably, the only study applying both an effective connectivity method optimized for resting-state fMRI and specification of nodes at a single-subject level reveals a very similar pattern of interaction in respect to the negative efferent connections of the PCC (Li et al., 2012).

DCM in general has a number of advantages compared to some other methods investigating effective connectivity (Friston, 2009, 2011; Penny et al., 2004; Roebroeck et al., 2011). It provides a more precise estimation at the neuronal level of how the rate of change of the hemodynamic response in one region influences the rate of change in another (Friston, 2009) and is robust and sensitive enough to examine clinical populations (Rowe et al., 2010). We applied spectral DCM in particular because it has been shown to be more accurate in identifying between-group effects than stochastic DCM (Razi et al., 2014) and in estimating effective connectivity (Li et al., 2011; Razi et al., 2014). Besides characterizing differences in directed connectivity strength, spectral DCM also allows to distinguish form and amplitude of neuronal fluctuations which may provide additional insight into the abnormalities underlying a particular disease (Friston et al., 2014).

We note that there is an imbalance of gender in our sample with a higher ratio of men to women in the patient groups compared to the control group. While this is often the case in research in disorders of consciousness, the potential interaction between gender and brain function after severe brain injury remains unexplored, and might require a larger sample size to be studied in detail. In addition, adjustment for gender has been investigated and found not to substantively alter the results.

In patients with severe head injury, processing of fMRI images is a critical issue. There is an ongoing debate on which method is the best to apply (Andersen et al., 2010; Ashburner and Friston, 2005; Crinion et al., 2007). All spatial normalization methods for group analyses have some disadvantages depending on the patients' type of brain lesions. This is especially problematic for patients such as the present cohort, in which etiology and brain lesions are highly heterogeneous including focal, wide-spread, and subtle lesions. For this reason, we applied affine-only normalization and carefully examined each processing step by visual inspection to avoid spurious results. Note that we extracted the eigenvariate of the time series using coordinates obtained by ICA at the single-subject level. This procedure ensures that, despite problems with normalization, the chosen nodes are functionally connected within each individual DMN network.

### 4.1 Conclusion

Taken together, this investigation shows that impaired consciousness is directly reflected in the imbalance between the inhibitory self-regulation of medial parietal regions and the strength of oscillations driving the neuronal responses. The PCC appears to have lost its control mechanisms by means of (self-) inhibitory regulation and, therewith, its function within the DMN as the main driven hub emphasizing the multifaceted and significant role of medial parietal regions in consciousness.

The following are the supplementary data related to this article.

Supplementary Table 2 Coordinates in Montreal Neurological Institute (MNI) space obtained from independent component analysis at the single-subject level.

Supplementary data to this article can be found online at http://dx.doi.org/10.1016/j.neuroimage.2015.01.037.
