# Supplementary material for: Impaired consciousness is linked to changes in effective connectivity of the posterior cingulate cortex within the default mode network
Source: Neuroimage. 2015 Apr 15;110:101–9. doi: 10.1016/j.neuroimage.2015.01.037 (PMC4389831; doi:10.1016/j.neuroimage.2015.01.037)
Supplement: Inline Supplementary Table S1 [file mmc1.doc]

**Table S1.** Patients’ information

| **Subjects** | **Age** | **Etiology** | **Time since onset**  **(in days)** | **Auditory function** | **Visual function** | **Motor function** | **Verbal / Oromotor function** | **Communication** | **Arousal** |
| --- | --- | --- | --- | --- | --- | --- | --- | --- | --- |
| *MCS* |  |  |  |  |  |  |  |  |  |
| MCS01 | 28 | Limbic encephalopathy | 148 | None | Visual pursuit | Flexion withdrawal | Oral reflexive movement | None | Without stimulation |
| MCS02 | 63 | Subarachnoidal hemorrhage | 62 | Auditory startle | Object localization: Reaching | Flexion withdrawal | None | None | Without stimulation |
| MCS03 | 62 | Subarachnoidal hemorrhage | 74 | Localization to sound | Visual pursuit | Flexion withdrawal | Oral reflexive movement | None | Attention |
| MCS04 | 46 | Multiple cerebral infarct | 41 | Auditory startle | Visual startle | Automatic motor response | Oral reflexive movement | None | With stimulation |
| MCS05 | 65 | Cardiopulmonary resuscitation | 85 | Localization to sound | Fixation | Object manipulation | Oral reflexive movement | None | Attention |
| MCS06 | 31 | Traumatic brain injury | 66 | None | None | Localization to noxious stimuli | Oral reflexive movement | None | With stimulation |
| MCS07 | 52 | Subarachnoidal hemorrhage | 146 | Localization to sound | Visual startle | Localization to noxious stimuli | Oral reflexive movement | None | Without stimulation |
| MCS08 | 61 | Intracerebral hemorrhage | 75 | None | Visual pursuit | Localization to noxious stimuli | Oral reflexive movement | None | With stimulation |
| MCS09 | 53 | Status epilepticus | 49 | None | None | Flexion withdrawal | Oral reflexive movement | None | Without stimulation |
| MCS10 | 51 | Status epilepticus | 135 | None | Visual pursuit | Flexion withdrawal | Oral reflexive movement | None | Without stimulation |
| MCS11 | 71 | Subarachnoidal hemorrhage | 70 | None | Visual pursuit | Flexion withdrawal | Oral reflexive movement | None | With stimulation |
| MCS12 | 32 | Multiple cerebral infarct | 37 | None | Visual pursuit | Abnormal posturing | Vocalization/Oral movement | None | With stimulation |
| *VS* |  |  |  |  |  |  |  |  |  |
| VS01 | 32 | Basilaris thrombosis | 922 | Auditory startle | None | Flexion withdrawal | Oral reflexive movement | None | With stimulation |
| VS02 | 66 | Traumatic brain injury & subarachnoidal hemorrhage | 82 | None | Visual startle | Flexion withdrawal | None | None | Without stimulation |
| VS03 | 36 | Cardiopulmonary resuscitation | 66 | Auditory startle | None | Abnormal posturing | Oral reflexive movement | None | Without stimulation |
| VS04 | 55 | Cardiopulmonary resuscitation | 82 | None | Visual startle | None | Oral reflexive movement | None | With stimulation |
| VS05 | 54 | Cardiopulmonary resuscitation | 70 | Auditory startle | Visual startle | None | None | None | With stimulation |
| VS06 | 35 | Status epilepticus | 49 | None | None | Flexion withdrawal | Oral reflexive movement | None | Without stimulation |
| VS07 | 59 | Subarachnoidal hemorrhage | 20 | Auditory startle | None | Flexion withdrawal | Oral reflexive movement | None | With stimulation |
| VS08 | 55 | Cardiopulmonary resuscitation | 186 | Auditory startle | Visual startle | Abnormal posturing | Oral reflexive movement | None | With stimulation |
| VS09 | 73 | Traumatic brain injury | 59 | None | None | Abnormal posturing | Oral reflexive movement | None | With stimulation |
| VS10 | 60 | Traumatic brain injury | 60 | None | None | Flexion withdrawal | Oral reflexive movement | None | With stimulation |
| VS11 | 43 | Cardiopulmonary resuscitation | 68 | Auditory startle | None | Abnormal posturing | Oral reflexive movement | None | Without stimulation |
| VS12 | 52 | Traumatic brain injury & status epilepticus | 64 | Auditory startle | Visual startle | Abnormal posturing | Vocalization/Oral movement | None | With stimulation |
| VS13 | 82 | Cardiopulmonary resuscitation | 27 | None | None | Abnormal posturing | Vocalization/Oral movement | None | Without stimulation |

MCS = patients in minimally conscious state; VS = patients in vegetative state (unresponsive wakefulness syndrome);
