# Supplementary material for: Impaired consciousness is linked to changes in effective connectivity of the posterior cingulate cortex within the default mode network
Source: Neuroimage. 2015 Apr 15;110:101–9. doi: 10.1016/j.neuroimage.2015.01.037 (PMC4389831; doi:10.1016/j.neuroimage.2015.01.037)
Supplement: Supplementary Table 2 — Coordinates in Montreal Neurological Institute (MNI) space obtained from independent component analysis at the single-subject level. [file mmc2.doc]

**Supplementary Table 2.** Coordinates in Montreal Neurological Institute (MNI) space obtained from independent component analysis at the single-subject level.

| **Subject** | **MFC** | **PCC** | **lIPL** | **rIPL** |
| --- | --- | --- | --- | --- |
| *Con* |  |  |  |  |
| CON1 | -2.51, 61.55, -16.24 | 4.68, -50.26, 15.99 | -40.27, -64.73, 47.33 | 50.48, -64.41, 45.75 |
| CON2 | -2.57, 61.03, -21.79 | 4.8, -48.26, 25.88 | -40.19, -64.11, 53.99 | 44, -64.43, 45.86 |
| CON3 | -2.48, 58.46, -14.81 | 1.52, -43.05, 24.3 | -46.75, -64.75, 47.44 | 71.79, -41.43, 26.31 |
| CON4 | 13.56, 60.57, -27.62 | 4.77, -51.76, 22.87 | -43.47, -64.43, 50.71 | 62.06, -41.47, 26.47 |
| CON5 | 4, 58.49, -14.92 | 1.52, -49.64, 22.71 | -40.19, -64.11, 53.99 | 55.85, -54.82, 44.71 |
| CON6 | -5.79, 57.93, -20.3 | 4.84, -57.83, 26.83 | -50.01, -61.58, 47.18 | 53.74, -57.71, 48.39 |
| CON7 | 3.98, 61.57, -16.35 | 4.82, -54.64, 26.51 | -50.01, -61.58, 47.18 | 47.28, -64.11, 49.14 |
| CON8 | -5.68, 65.35, -9.84 | 4.67, -47.06, 15.68 | -55.47, -48.94, 44.9 | 53.71, -61.21, 45.38 |
| CON9 | -2.53, 61.34, -18.46 | 4.81, -51.45, 26.2 | -46.75, -64.75, 47.44 | 50.48, -64.41, 45.75 |
| CON10 | -2.62, 47.54, -28.3 | -1.78, -53.36, 17.53 | -43.47, -64.43, 50.71 | 50.48, -64.41, 45.75 |
| CON11 | -2.42, 68.76, -7.99 | 1.56, -48.27, 25.94 | -46.72, -61.25, 50.46 | 55.85, -54.82, 44.71 |
| CON12 | 4.1, 62.51, -6.36 | 4.74, -43.25, 22.02 | -55.44, -52.02, 46.33 | 58.83, -44.67, 26.85 |
| *MCS* |  |  |  |  |
| MCS1 | -8.41, 48.35, 26.63 | 4.71, -46.75, 19.01 | -40.17, -67.3, 54.3 | 47.31, -54.02, 53.74 |
| MCS2 | 3.88, 54.26, -25.71 | 1.59, -48.06, 28.16 | -65.46, -43.29, 25.46 | 50.48, -64.41, 45.75 |
| MCS3 | 7.21, 64.78, -16.72 | 7.99, -46.43, 22.28 | -50.02, -58.38, 46.87 | 53.71, -61.21, 45.38 |
| MCS4 | 4.83, 32.94, 44.74 | 1.52, -43.05, 24.3 | -46.76, -61.56, 47.12 | 50.48, -64.41, 45.75 |
| MCS5 | -15.4, 65.32, -9.67 | 4.83, -48.05, 28.1 | -37.04, -61.53, 46.96 | 47.24, -64.42, 45.81 |
| MCS6 | 3.86, 57.45, -26.02 | 1.55, -45.08, 25.62 | -65.39, -38.41, 31.7 | 53.46, -41.18, 29.95 |
| MCS7 | 10.32, 60.56, -27.56 | 1.59, -48.06, 28.16 | -52.46, -41.87, 28.46 | 44.02, -57.74, 48.56 |
| MCS8 | -1.89, 58.47, 31.12 | 7.97, -53.12, 19.58 | -65.46, -43.29, 25.46 | 53.68, -45.05, 46.02 |
| MCS9 | 10.38, 47.9, -25.19 | 8.01, -51.75, 22.81 | -37.04, -61.53, 46.96 | 55.86, -58.01, 45.03 |
| MCS10 | 3.84, 60.53, -27.45 | 4.84, -57.83, 26.83 | -40.27, -64.73, 47.33 | 59.09, -54.81, 44.65 |
| MCS11 | 7.73, 54.48, 22.39 | 4.75, -46.44, 22.34 | -43.8, -45.03, 28.63 | 53.71, -61.21, 45.38 |
| MCS12 | -15.37, 65.53, -7.45 | 4.8, -48.26, 25.88 | -46.76, -61.56, 47.12 | 55.86, -58.01, 45.03 |
| *VS* |  |  |  |  |
| VS1 | 13.76, 68.61, -10.49 | 8.07, -48.04, 28.04 | -43.47, -64.43, 50.71 | 44.04, -64.12, 49.19 |
| VS2 | 7.92, 47.11, 35.44 | 11.32, -57.81, 26.71 | -40.19, -64.11, 53.99 | 40.75, -61.26, 45.6 |
| VS3 | 3.89, 34.7, -28.26 | -1.65, -57.85, 26.94 | -40.27, -64.73, 47.33 | 50.47, -67.81, 43.85 |
| VS4 | 4.58, 61.68, 30.69 | 4.82, -44.86, 27.78 | -40.19, -64.11, 53.99 | 50.47, -67.81, 43.85 |
| VS5 | -5.83, 41.25, -26.5 | -1.69, -39.56, 27.37 | -43.51, -64.74, 47.38 | 53.71, -61.21, 45.38 |
| VS6 | 4.61, 46.58, 29.95 | 4.82, -54.64, 26.51 | -43.51, -64.74, 47.38 | 44.04, -64.12, 49.19 |
| VS7 | 4.14, 70.47, -1.54 | -1.7, -36.37, 27.06 | -40.29, -58.35, 46.7 | 47.23, -54.64, 47.08 |
| VS8 | 13.56, 60.57, -27.62 | 4.82, -54.64, 26.51 | -40.27, -64.73, 47.33 | 47.23, -61.23, 45.49 |
| VS9 | -5.79, 31.68, -25.55 | 1.59, -48.06, 28.16 | -40.19, -64.11, 53.99 | 50.47, -67.81, 43.85 |
| VS10 | 4.53, 61.37, 27.36 | 1.57, -44.87, 27.84 | -43.47, -64.43, 50.71 | 47.2, -54.85, 44.86 |
| VS11 | 4.65, 53.48, 34.87 | 4.84, -57.83, 26.83 | -33.81, -58.33, 46.58 | 50.55, -60.6, 52.1 |
| VS12 | -5.62, 69.06, -4.6 | 1.59, -48.06, 28.16 | -43.51, -64.74, 47.38 | 47.24, -64.42, 45.81 |
| VS13 | -2.57, 61.03, -21.79 | 4.73, -53.13, 19.64 | -43.51, -64.74, 47.38 | 53.71, -61.21, 45.38 |

CON = control subjects; MCS = patients in minimally conscious state; VS = patients in vegetative state (unresponsive wakefulness syndrome);
